# Mode of Delivery and Its Influence on the Acquisition of *Streptococcus mutans* in Infants

**DOI:** 10.5005/jp-journals-10005-1386

**Published:** 2016-12-05

**Authors:** Ritu G Ubeja, Chetan Bhat

**Affiliations:** 1 Postgraduate Student, Department of Pediatric and Preventive Dentistry, Bharati Vidyapeeth Dental College and Hospital, Pune, Maharashtra India; 2 Associate Professor, Department of Pediatric and Preventive Dentistry, Bharati Vidyapeeth Dental College and Hospital, Pune, Maharashtra India

**Keywords:** Cesarean section, Normal delivery, Oral micro-biota, *Streptococcus mutans.*

## Abstract

**Introduction:**

Dental caries pose distinct challenges when it comes to determining their microbial etymology. *Streptococcus mutans* play an important role in dental caries. The aim of the present study was to compare oral microbiota in infants delivered by these different routes. A study was conducted on 40 infants. Swab sample collection was done for the detection of S. *mutans.* Our study indicated no differences in oral microbiota in infants due to mode of delivery.

**Aim:**

To assess whether infants born through cesarean section delivery or infants born through normal delivery influence the initial acquisition of S. *mutans* in infants.

**Settings and design:**

The study was carried out on the premises of Bharati Hospital, Pune, wherein 40 infants (3-36 months) were enrolled for the study. Two groups were designed.

*Group I:* Infants born with cesarean section delivery

*Group II:* Infants born with normal section delivery

**Materials and methods:**

Bacterial swab sampling was done in the participants for the detection of S. *mutans.* Colony-forming units on each plate were determined for the estimation of *S. mutans* level in oral cavity.

**Statistical analysis used:**

Bar diagram analysis and chi-square test were performed to derive p-value.

**Results:**

The p value derived at the end of the study was 0.52. Hence, analysis of data demonstrates no significant influence of cesarean section delivery and normal delivery on oral microbiota development in infants.

**Conclusion:**

Initial acquisition of oral S. *mutans* in infants is not dependent on the mode of delivery.

**Key Messages:**

Initial acquisition of S. *mutans,* Mode of delivery.

**How to cite this article:**

Ubeja RG, Bhat C. Mode of Delivery and Its Influence on the Acquisition of *Streptococcus mutans* in Infants. Int J Clin Pediatr Dent 2016;9(4):326-329.

## INTRODUCTION

During and shortly after birth, various bacterial species colonize the epithelial surfaces in the oral cavity of bac-terially naive infants.^1^ In vaginally delivered infants, the first exposure to microorganisms occurs during passage through the birth canal, whereas in infants born through cesarean section (henceforth referred to as C-section in the paper), the first exposure to bacteria is from the skin of parents and health providers, and medical equipment.^2^ Mutants Streptococci were detected more frequently and at a younger age in the oral cavity of children delivered by C-section than those delivered vaginally.^1^ *Streptococcus mutans* transmission in the oral cavity of the children is more frequent from the saliva of mother who infects the child during her care, especially if she herself does not maintain oral hygiene, lacks treatment for caries in her oral cavity, and basic hygiene rules are neglected.^3^

A prominent source from where bacteria might be acquired by newborn infant is the parturient canal. Vaginally delivered infants offer oral bacteria in less hospitable environment. They develop more resistance to these bacteria in their first year of life, in part because of exposure to a greater variety and intensity of bacteria from their mothers and the surrounding environment at birth. C-section babies have less bacterial exposure at birth and therefore show less resistance.^4^

The aim of our study is to assess whether infants born through cesarean section delivery or infants born through normal vaginal delivery influence the initial acquisition of *S. mutans* in infants.

## MATERIALS AND METHODS

Case history sheets was taken from the participating mothers, who then signed informed consent at recruitment. The mothers completed a questionnaire on other possible confounders, such as health issues (allergy, infections, or stomach problems). Saliva was chosen as the study specimen in the predentate rather than plaque, as saliva frequently reflects the overall oral flora and serves as a reservoir for any tooth-associated species. Thus the variety of ecological sites to be indirectly assessed consequently increases with the effect of presence of teeth. The procedures followed were in accordance with the ethical guidelines laid down by the Helsinki Declaration and the Ethical Committee at Bharati Vidyapeeth Dental College and Hospital, Pune.

Forty infants (3-36 months) were enrolled for the study. Two groups were designed (Flow Chart 1)

*Group I:* Infants born with cesarean section delivery

*Group II:* Infants born with normal section delivery.

**Flow Chart 1:**
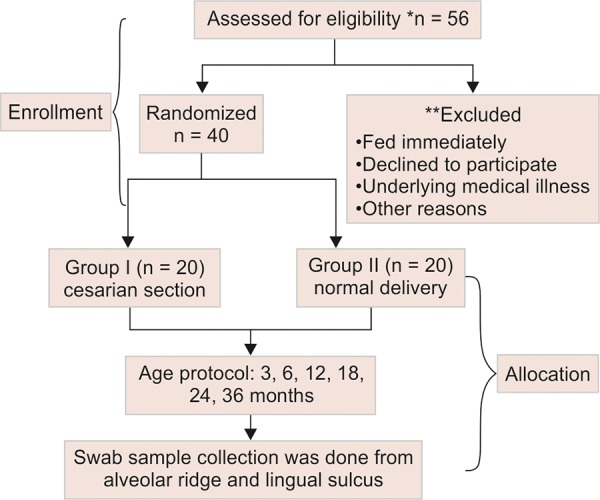
Schematic representation of the study, from infant enrollment to swab sampling

### Bacterial Sample Collection

**Fig. 1:**
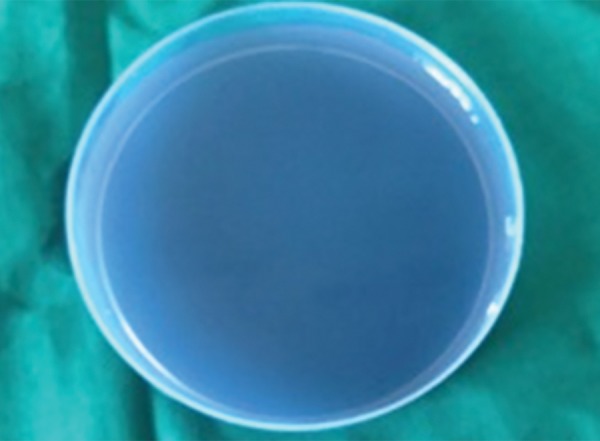
Mitis salivarius-bacitracin sucrose agar plate used for study

**Fig. 2:**
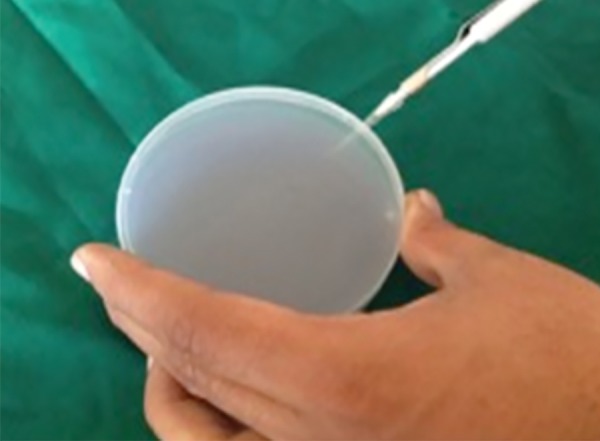
The solution pipetted onto mitis salivarius-bacitracin sucrose agar plate for further incubation and study of S. *mutans* colonies

Swab sampling was done in the participants for detection of *S. mutans* with SalivaBio Infant’s Swab (SIS),^5^ Biogenuix Medsystems Pvt Ltd, New Delhi, India. Sufficient saliva is usually absorbed in few minutes. All the samples were placed in the sterile plastic test tube and processed within 1 hour of the collection of sample. The swab was placed in 1 mL of 0.5 M phosphate buffer (pH: 7) solution prepared by mixing the buffer powder in distilled water and was vortexed for 1 minute. The sample was diluted in the ratio 1:10 with the phosphate buffer solution and was then vortexed. A 50-μL volume of each dilution was pipetted onto each mitis salivarius-bacitracin sucrose agar plate^6-9^ (Fig. 1) and evenly distributed using sterile spreaders for the cultivation of *S. mutans.* The plates were incubated at 37°C for 72 hours, and the number of colonies was counted based on the colony character. The colonies had crusted glass appearance and colony-forming units on each plate were numerated for the estimation of *S. mutans* level in the oral cavity (Fig. 2).

## RESULTS

The saliva sample was collected from infants who are 3, 6, 12, 18, 24, and 36 months and the numbers participating at different age groups was tabulated (Table 1).

**Graph 1:**
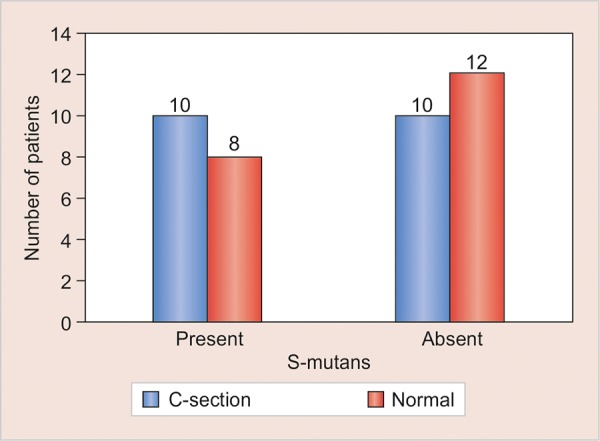
Relative occurrence of S. *mutans* in C-section and normal mode of delivery (C-section = Cesarean section)

**Graph 2:**
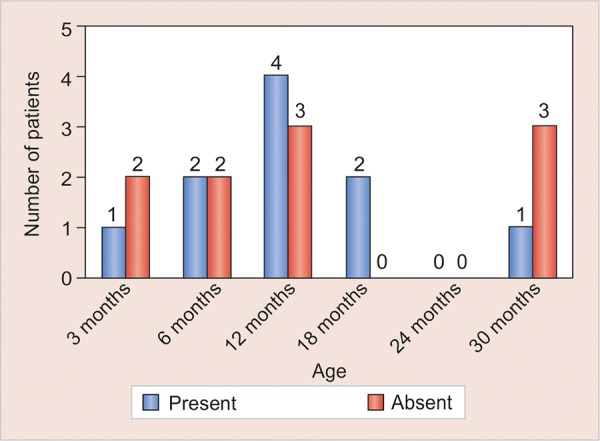
Occurrence of *S. mutans* in C-section delivery relative to infant age (C-section = Cesarean section)

**Graph 3:**
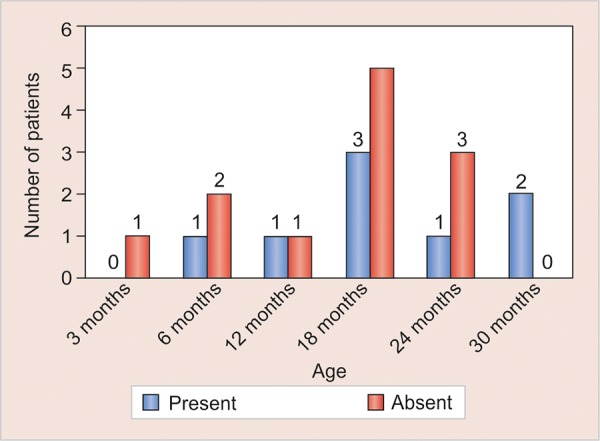
Occurrence of S. *mutans* in normal delivery relative to infant age

In Tables 2 and 3, the number of infants in whom *S. mutans* was present and absent in different mode of deliveries was tabulated. Relative occurrence of *S. mutans* in C-section and normal mode of delivery was derived (Graph 1). Statistical analysis in infants was done separately for C-section and normal delivery with the help of bar diagrams (Graphs 2 and 3).

Chi-square test is used and the p value obtained is 0.52; hence the results were not statistically significant (Table 4).

The results derived demonstrate no significant influence of cesarean section delivery and normal delivery on occurrence of *S. mutans* in infants.

**Table 1:** **Table 1**: Samples of 40 infants aged 3 to 36 months, classified into two groups. Group I denotes sampling of 20 infants delivered through cesarean section. Group II denotes sampling of 20 infants delivered through normal delivery mode

*Age*		*Group I (n = 20)* *Cesarean* *section delivery*		*Group II (n = 20)* *Normal delivery*	
3 months		3		1	
6 months		4		3	
12 months		7		2	
18 months		2		8	
24 months		0		4	
36 months		4		2	

**Table 2:** **Table 2:** Classification of occurrence of S. *mutans* in infants delivered through C-section

		C-section* delivery *-S. mutans occurrence*					
*Age*		*Present*		*Absent*		*Total*	
3 months		0		1		1	
6 months		1		2		3	
12 months		1		1		2	
18 months		3		5		8	
24 months		1		3		4	
36 months		2		0		2	

*C-section = Cesarean section

**Table 3:** **Table 3:** Classification of occurrence of S. *mutans* in infants delivered through normal delivery

		*Normal delivery -*S. *mutans occurrence*					
*Age*		Present*		*Absent*		*Total*	
3 months		1		2		3	
6 months		2		2		4	
12 months		4		3		7	
18 months		2		0		2	
24 months		0		0		0	
36 months		1		3		4	

**Table 4:** **Table 4:** Infants with S. *mutans* presence and absence in normal and C-section delivery categories. The resulting p-value of the study derived is 0.52, which is statistically insignificant

		*Mode of delivery*					
S. *mutans*		C-section*		*Normal*		*p-value*	
Present		10		8		0.52	
Absent		10		12			
Total		20		20			

*C-section = Cesarean section

## DISCUSSION

This study emphasizes on the mode of delivery affecting establishment of *S. mutans* in the oral cavity of infants, which is the causative organism for the dental caries so that early intervention can be taken. The immune defense system of a newborn is reinforced by extensive exposure to maternal microorganisms through birth canal; thus, it was speculated that birth by cesarean section would have an impact on infant’s oral colonization of *S. mutans.^2^*

Saliva rather than plaque was chosen as the study specimen in the predentate as the saliva is frequently taken as reflection of overall oral flora and serve as reservoir for any tooth-associated species, thus promoting the effect of teeth, consequently increasing the variety of ecological sites to be indirectly assessed.^9-12^

Higher numbers of taxa were detected among infants delivered vaginally, compared with those delivered by C-section, with probes to the 16S rRNA gene of cultivated and uncultivated oral bacteria in a microarray format.^7^ The socioeconomic status of the family appeared to be an important factor in the early colonization of micro-organism.^8^ Isenberg et al reported that cesarean-born children had significantly decreased numbers of bacterial species and colony-forming units than vaginally delivered children, suggesting that by avoiding passage through birth canal, C-section infants may be less likely to be exposed to various bacterial species and strains from the mothers.^13^

This can be attributed to the awareness of the mothers with high socioeconomic status and education concerning feeding and oral hygiene practices of their infants.

## CONCLUSION

This study supports the premise that mode of delivery does not correlate the early colonization of *S. mutans* in the oral cavity of infants from 3 to 36 months.
